# Pathway-Based Mendelian Randomization for Pre-Infection IL-6 Levels Highlights Its Role in Coronavirus Disease

**DOI:** 10.3390/genes15070889

**Published:** 2024-07-06

**Authors:** Zoha Kamali, Nafiseh Esmaeil, Chris H. L. Thio, Ahmad Vaez, Harold Snieder

**Affiliations:** 1 Department of Bioinformatics, Isfahan University of Medical Sciences, Isfahan 81746-73441, Iran; 2 Department of Epidemiology, University of Groningen, University Medical Centre Groningen, Hanzeplein 1 (9713 GZ), P.O. Box 30.001, 9700 RB Groningen, The Netherlandsh.snieder@umcg.nl (H.S.); 3 Department of Immunology, School of Medicine, Isfahan University of Medical Sciences, Isfahan 81746-73441, Iran; n_esmaeil@med.mui.ac.ir; 4 Research Institute for Primordial Prevention of Non-Communicable Disease, Isfahan University of Medical Sciences, Isfahan 81746-73441, Iran

**Keywords:** severe COVID-19, interleukin 6, GWAS, mendelian randomization, causality

## Abstract

Objectives: Interleukin 6 (IL-6) levels at hospital admission have been suggested for disease prognosis, and IL-6 antagonists have been suggested for the treatment of patients with severe COVID-19. However, less is known about the relationship between pre-COVID-19 IL-6 levels and the risk of severe COVID-19. To fill in this gap, here we extensively investigated the association of genetically instrumented IL-6 pathway components with the risk of severe COVID-19. Methods: We used a two-sample Mendelian randomization study design and retrieved genetic instruments for blood biomarkers of IL-6 activation, including IL-6, soluble IL-6 receptor, IL-6 signal transducer, and CRP, from respective large available GWASs. To establish associations of these instruments with COVID-19 outcomes, we used data from the Host Genetics Initiative and GenOMICC studies. Results: Our analyses revealed inverse associations of genetically instrumented levels of IL-6 and its soluble receptor with the risk of developing severe disease (OR = 0.60 and 0.94, respectively). They also demonstrated a positive association of severe disease with the soluble signal transducer level (OR = 1.13). Only IL-6 associations with severe COVID-19 outcomes reached the significance threshold corrected for multiple testing (*p* < 0.003; with COVID-19 hospitalization and critical illness). Conclusions: These potential causal relationships for pre-COVID-19 IL-6 levels with the risk of developing severe symptoms provide opportunities for further evaluation of these factors as prognostic/preventive markers of severe COVID-19. Further studies will need to clarify whether the higher risk for a severe disease course with lower baseline IL-6 levels may also extend to other infectious diseases.

## 1. Introduction

Coronavirus disease 2019 (COVID-19) has led to more than seven million deaths worldwide since its emergence in late 2019 [1]. Many efforts have been invested in identifying appropriate drug targets for severe forms of the disease [2,3,4]. Among these, interleukin 6 (IL-6) antagonists, including tocilizumab and sarilumab, have been suggested for the treatment of patients with severe COVID-19 [5]. A recent meta-analysis of clinical trials reported the protective effect of anti-IL6 therapy against hospitalization but not against developing severe symptoms or the need for mechanical ventilation [6]. In terms of prognosis, recent studies have shown the value of IL-6 in the prediction of severe COVID-19 at hospital admission [7,8]. However, less is known about pre-COVID-19 levels of IL-6 and their possible causal contribution to disease outcome. One possible way of obtaining more knowledge on the topic is by studying genetic variants associated with IL-6 levels, or related signaling components, and their effects on COVID-19 outcomes. An example is rs1800795 on the *IL-6* gene; the G allele of this variant is associated with lower IL-6 levels [9] and also with a higher risk of severe COVID-19 [10]. Meanwhile, other negative reports exist that fail to establish an association for *IL-6* promoter SNPs with COVID-19 severity [11].

**Figure 1:**
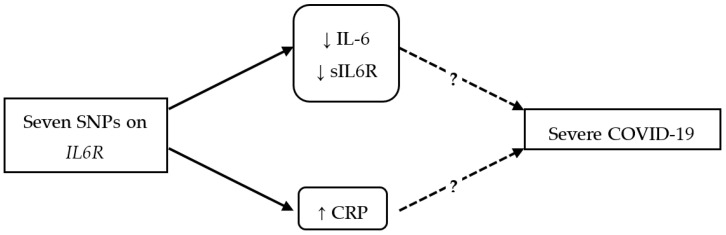
The effects of seven IL6R SNPs influencing IL6R splicing on CRP, as well as other IL-6 pathway components, i.e., IL-6 and its soluble receptor levels. Up and down arrows show positive and negative effects, respectively. The exclusion restriction and independence assumptions of MR might be violated here.

The Mendelian randomization (MR) approach uses genetic variants as instrumental variables to minimize bias due to confounding. MR has three key assumptions: (1) the instrument is strongly related to the exposure (relevance), (2) the instrument is not related to any confounder in the exposure–outcome relation (independence), and (3) the instrument only affects the outcome through the exposure (exclusion restriction) [12]. Under these assumptions, MR yields estimates unconfounded by factors such as background diseases and concomitant treatments. Given the lack of sufficiently strong genetic instruments for IL-6, two recent studies have used seven single-nucleotide polymorphisms (SNPs), on or near *IL6R*, associated with C-reactive protein (CRP) as instrumental variables (IVs), and these showed significant causal associations with COVID-19 susceptibility, hospitalization, and severity [13,14]. However, the authors acknowledged the association of these seven SNPs with IL-6 and its receptor, and hence, the observed associations with COVID-19 outcomes may (also) be driven by the effects of these SNPs on the soluble/membranous receptor level and, consequently, IL-6 levels [13] (Figure 1), rather than CRP itself [15].

**Figure 2:**
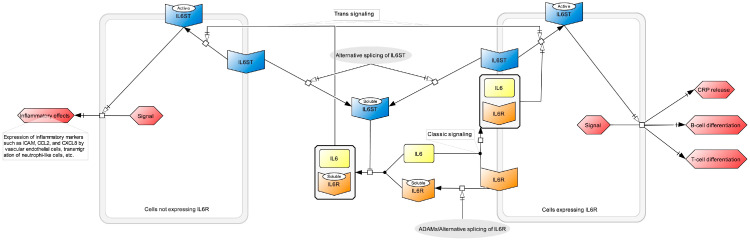
A simplified schematic of the known relationships between IL-6 signaling components investigated in this study, i.e., IL6, IL6R, IL6ST, and CRP. Systems Biology Graphical Notation (SBGN) was used to generate the figure. The information in this figure was inferred from references [16,17,18,19,20]. Please note that elevated IL-6 and IL6R, as independent variables in this model, are expected to increase both classic and trans signaling and, hence, all output phenotypes, including CRP levels. However, assuming a constant reservoir of the IL6R transcript, appropriate splicing (the absence of a gray oval, which triggers the conversion of IL-6 receptor to its soluble form) may result in a reduced soluble IL6R level and increased membrane-bound receptors. Hence, elevated classic signaling and CRP release are expected, while IL-6 level in blood is reduced because of binding to membrane-bound receptors in higher levels (Figure 1).

To address these gaps and uncertainties, in this current study, we have focused on different IL-6 signaling components and used the latest and largest available GWASs to extract instrumental variables for each. Then, through an extended MR study design, we sought to strengthen the evidence for a causal relation of blood levels of IL-6 pathway components, including IL-6, sIL6R, soluble IL6ST (also called gp130), and CRP (as shown in Figure 2), on COVID-19 outcomes. Furthermore, we used multivariable MR to ascertain the main driver(s) of possible associations, as well as other sensitivity analyses to confirm the findings.

## 2. Methods

### 2.1. COVID-19 GWAS Data

**Figure 3:**
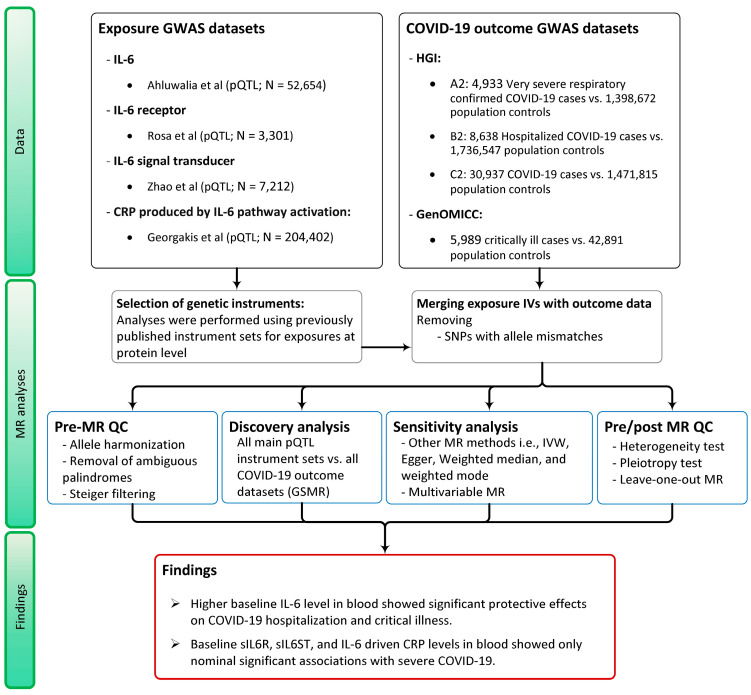
Flow diagram of the study methods and datasets used. sIL6S T: soluble IL-6 signal transducer; CRP: C-reactive protein; GSMR: generalized summary-data-based Mendelian randomization. HGI: host genetics initiative; GenOMICC: Genetics of Susceptibility and Mortality in Critical Care.

Genome-wide association study (GWAS) data on severe COVID-19 from the COVID-19 Host Genetic Initiative consortium, release 4, were used; these were taken from 4933 laboratory-confirmed SARS-CoV-2 patients who were hospitalized and required respiratory support or died and were compared with 1,398,672 controls (HGI_ALL_A2) [21]. Other COVID-19 outcomes such as hospitalized (HGI_ALL_B2) or positive COVID-19 (HGI_ALL_C2) patients vs. population controls were also used, for which details are listed in Figure 3, as well as Table S1. The results of whole-genome sequencing of over 5989 critical COVID-19 cases by the Genetics of Susceptibility and Mortality in Critical Care (GenOMICC) study was used as a fourth dataset [22].

### 2.2. Genetic Instruments for IL-6 Signaling

For IL-6, we used three SNPs from a recent study of 52,654 individuals [23]. For the soluble IL-6 receptor (sIL6R), we used 34 independent genetic variants (r^2^ < 0.1) associated with blood sIL6R levels in 3301 European individuals, as suggested by Rosa et al. [24] (F-statistic > 15, with F being calculated as beta^2^/se^2^). For the soluble IL-6 signal transducer (sIL6ST), we used three SNPs as genetic instruments at the protein level, as provided by Zhao and colleagues [25]. For CRP levels resulting from the IL-6 pathway, we used seven SNPs in the *IL6R* gene, as suggested by Georgakis and colleagues [15]. All SNPs used as instruments in MR analyses and their association summary statistics are listed in Table S2. Information on the original GWASs is also available in Table S1.

### 2.3. Analysis Approach

Prior to MR, ambiguous palindromic SNPs were excluded to avoid errors due to incorrectly matched alleles; for palindromes with a minor allele frequency of <0.3, we inferred the correct strand. We also applied Steiger filtering, by default settings, to avoid violation of exclusion restriction through reverse causality (Pre-MR QC).

We used a recently introduced powerful MR approach, Generalized Summary-data-based Mendelian Randomization (GSMR) [26], version 1.0.9. This method corrects for the uncertainty in SNP exposure effects (in addition to the uncertainty in SNP outcome effects), accounts for the residual LD between instruments, and excludes potentially pleiotropic SNPs through the HEIDI outlier test (*p*-value < 0.01). For exposures with fewer than 5 instruments, the HEIDI outlier test was not performed, as in this scenario, the test is unreliable. Instead, we used two additional steps for these exposures: (1) searching their SNP effect estimates in other independent GWASs [27,28] to avoid ‘Winner’s curse’ and (2) leave-one-SNP-out MR to visually inspect the presence of influential, possibly pleiotropic SNPs.

MR sensitivity analyses were performed, including the inverse-variance weighted (IVW) method and MR methods which are robust to varying degrees of violation of exclusion restriction criterion (i.e., horizontal pleiotropy) i.e., MR Egger [29], weighted median [30], and weighted mode [31], all implemented in the *TwoSampleMR* R package [32], version 0.5.5. In addition, we performed multivariable MR [33] using the same package, in which we modelled CRP, IL-6, and sIL6R simultaneously to correct for potential horizontal pleiotropy and to ascertain the primary driver of any observed association. All original exposure GWASs were used here as well, except for IL-6 for which the original GWAS was not publicly available. Therefore, we used the replication IL-6 GWAS [27] for this analysis. Additionally, we assessed potential bias due to pleiotropy by inspecting heterogeneity and directional pleiotropy tests as post-MR quality checks (Post-MR QC). The analysis pipeline is depicted in Figure 3.

Altogether, we conducted a total of 16 tests at discovery (4 instrument sets × 4 COVID-19 outcome datasets). Hence, we consider a conservative Bonferroni-corrected *p*-value < 0.05/16, i.e., 0.003 as significant in our MR analyses. We also conducted a total of 16 tests as sensitivity analyses. For clarity and robustness, we abided by the Strengthening the Reporting of Observational Studies in Epidemiology (STROBE) guidelines for MR studies [34].

## 3. Results

### 3.1. IL-6

**Figure 4:**
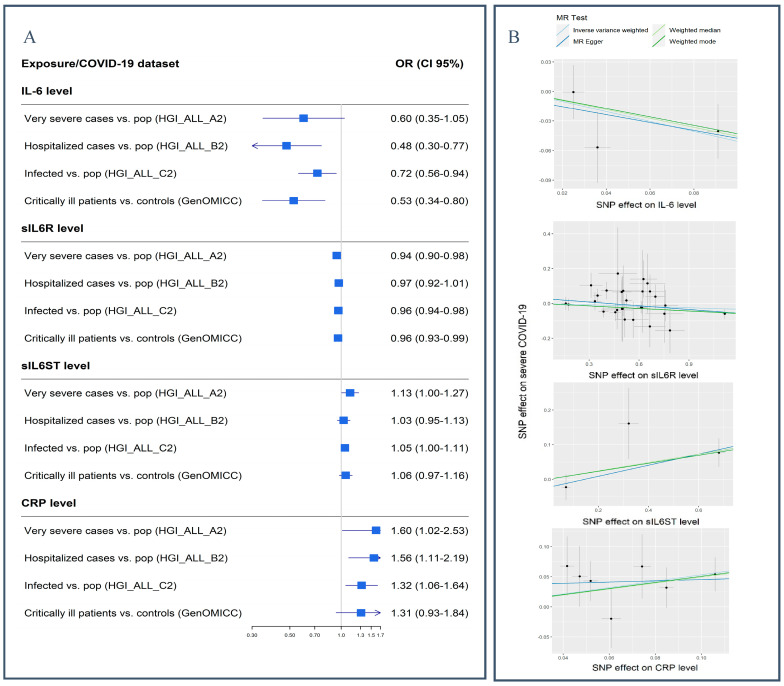
MR results of different IL-6 components, as exposures, against COVID-19 outcomes from different datasets. (**A**) Forest plot of GSMR estimates based on all SNP effects for each exposure. (**B**) Scatter plots showing SNP effects on exposures, as well as on severe COVID-19 (HGI_A2 dataset). IL-6: interleukin 6; sIL6R: soluble IL-6 receptor; sIL6ST: soluble IL-6 signal transducer; CRP: C-reactive protein.

The most significant association of genetically instrumented IL-6 was observed with COVID-19 hospitalization, i.e., HGI_ALL_B2 (OR (CI 95%) = 0.48 (0.30–0.76), *p* = 0.002) (Figure 4, Table S3). It also showed a significant association with critical COVID-19, i.e., the GenOMICC dataset, at *p* < 0.003, with a similar effect direction (OR (CI 95%) = 0.53 (0.34–0.80)). Sensitivity analysis confirmed the association with a consistent direction of effect across all methods and significant results in two of them, namely IVW and weighted median (Table S3B).

There was borderline heterogeneity (*p* = 0.07), but there was no evidence for directional horizontal pleiotropy (*p* = 0.72). Furthermore, a leave-one-out analysis did not reveal influential, potentially pleiotropic, SNPs (Figure S1). All three SNPs showed a consistent direction of effects on IL-6 using another independent GWASs (N ~ 30k) [27] (Table S5).

### 3.2. IL-6 Receptor

sIL6R showed a suggestive modest negative effect on COVID-19 outcomes (ORs ranging 0.94 to 0.97) (Figure 4), but these did not reach statistical significance, except for COVID-19 infection, i.e., HGI_ALL_C2 (*p* = 0.0005).

### 3.3. IL-6 Signal Transducer

The genetically instrumented IL-6 signal transducer showed a suggestive positive association with severe COVID-19 (OR (CI 95%) = 1.13 (1.00–1.27), *p* = 0.04) (Figure 4). Although robust to sensitivity analysis, this association did not reach statistical significance.

### 3.4. CRP

The genetically instrumented CRP showed only nominally significant associations, with ORs ranging from 1.31 to 1.60 for different COVID-19 datasets (Figure 4, Table S3). None of these reached our statistical significance threshold of 0.003. Moreover, multivariable MR did not return a significant association for CRP (*p* > 0.5) in the presence of IL-6 (*p* = 0.05) and sIL6R (*p* = 0.13) (Table S6).

The STROBE-MR [34] checklist is presented in Table S7.

## 4. Discussion

As the first Mendelian randomization study examining the four key IL-6 pathway components together, we found evidence for the protective effect of higher baseline IL-6 levels against developing severe COVID-19. Soluble IL-6 receptor levels showed an effect that was directionally consistent with the effect of IL-6, although this did not reach our conservative, Bonferroni-corrected significance threshold. Higher levels of the soluble form of the IL-6 signal transducer were associated with higher odds of developing severe symptoms, although this was only nominally significant. The same direction of association, with a nominally significant *p*-value, was observed for C-reactive protein. Our results suggest a higher baseline IL-6 reservoir protecting against developing severe COVID-19, as discussed below.

The inverse association between pre-COVID-19 IL-6 levels and COVID-19 hospitalization/critical illness is in line with the current literature, suggesting the overall protective role of higher IL-6 levels, providing immune readiness [35,36]. The presence of IL-6 is necessary for the differentiation and maturation of follicular T helper cells (Tfh), which play a critical role in optimal anti-viral antibody production [37]. IL-6 also increases the IgG production by B cells. Therefore, previously elevated levels of IL-6 probably support the timely differentiation of Tfh cells and the production of protective antibodies [38]. Furthermore, IL-6 together with IL-15 regulates the cytotoxic function of the CD8+ T cells as the main adaptive immune cells in viral infections [39].

In vitro studies have shown that IL-6 acts in a negative feedback loop that decreases or terminates immune responses [35]. Also, studies in mice demonstrate how baseline IL-6 can diminish pro-inflammatory cytokines released after aerosol exposure to endotoxin [40]. Therefore, the overexpression of IL-6 prior to infection may induce this negative loop before it leads to a cytokine storm. Considering that previous studies have shown contradictory roles—protective and adverse—for IL-6 in viral lung infections [41,42], it is possible that the effect of IL-6 on COVID-19 has a protective or pathogenic role depending on cytokine levels before and during the disease. Accordingly, a serial evaluation of IL-6 levels might be helpful in predicting COVID-19 pathogenesis.

Higher baseline IL-6 levels can also produce a rapid immune response through the antiviral activity of IL-6-dependent interferons type I (IFNα and IFNβ) [43,44]. Genetically downregulated IFN levels have been related to the development of severe COVID-19 symptoms [45]. IL-6 can amplify the IFN response by inducing/increasing its intermediate signal processors such as STATs [43]. Future studies are needed to investigate this potential pathway.

Furthermore, we observed a small protective effect for higher soluble IL-6 receptor levels in blood against severe COVID-19. This may imply a booster effect on IL-6′s protective role against the virus for sIL6R at baseline, possibly through trans-signaling [36]. IL-6 trans-signaling participates in the rapid generation and activation of CD8+ T cells [46]. Peters et al. assessed the role of sIL-6R in hslL-6R transgenic mice and found that the half-life of IL-6 was significantly elongated, and its serum levels were elevated. They showed how, in the presence of sIL-6R, animals are more sensitive to IL-6, and low levels of IL-6 can stimulate target cells [47]. However, given that this effect was not statistically significant (0.003 < *p* < 0.05), some caution is warranted.

A recent GWAS in a Chinese population (ncase = 632, ncontrol = 3021) reported an IL-6 down-regulating SNP in patients (rs2069837) to be protective against severe COVID-19 (OR = 0.41 for the G allele) [48], contradicting our results. However, this finding was not replicated in the latest release of the HGI consortium with a larger sample size (East Asians, ncase = 794, ncontrol = 4862), as it shows only a nominally significant *p*-value of 0.01 and an inconsistent direction of effect (β = 0.2, i.e., OR = 1.2). In the European population of the same resource, there was no significant association for rs2069837 and severe COVID-19 (*p*-value = 0.2).

Higher IL-6 signal transducer levels, in soluble form, show a suggestive causal effect on severe COVID-19 (0.003 < *p* < 0.05). This may reflect its inhibitory role on the formation of the IL-6:sIL6R complex (Figure 2) and, therefore, trans signaling [18]. Lamerts et al. [49]. found that IL-6 classic signaling is also suppressed by soluble IL6ST through its interaction with membrane-bound IL-6:IL6R complexes. Moreover, assuming constant *IL6ST* gene expression, the elevation of soluble IL6ST due to *IL6ST* SNPs means a reduction in membrane-bound IL6ST and, therefore, lower classical signaling (see the alternative splicing of IL6ST in Figure 2).

Therefore, from our combined findings on IL-6, IL6R, and IL6ST, it can be concluded that a higher baseline level of IL-6 has protective effects against COVID-19, and in the presence of higher levels of IL-6 signaling inhibitors, like soluble IL6ST, a delay in the effective immune response leads to the exacerbation of the disease.

We also observed nominally significant effects of CRP levels on severe COVID-19. However, this association could also be driven by horizontal pleiotropy, i.e., SNP effects on the soluble/membranous receptor level and, consequently, IL-6 level [13] (Figure 1), knowing that the former has other diverse roles not going through CRP, as discussed earlier. To distinguish between the true effect of CRP and possible horizontal pleiotropy, we performed multivariable MR. This did not show a significant association between CRP and severe COVID-19 (*p* > 0.5) when adjusted for sIL6R and IL-6, suggesting that there was indeed horizontal pleiotropy.

The strengths of our study include the use of different IV sets, all focused on IL-6 signaling components, different large-scale genetic data for exposures and outcomes, and a variety of MR methods. We also examined the robustness of results through different post-MR QC tests. Also, to avoid selection bias, we focused our MR study on larger COVID-19 GWASs with population controls, rather than those with fewer precisely matched controls. We were limited to using genetic instruments for whole-blood levels of IL-6 traits, given the current lack of large-scale GWASs, and, hence, genetic instruments for IL-6 traits in tissues that are possibly more relevant to COVID-19 (e.g., lung tissue). Finally, no genetic instruments for IL-6 from ancestries other than European were currently available, limiting the generalizability of our findings.

Based on our findings here, and despite the known effects of IL-6 levels during-COVID-19, higher levels of pre-COVID-19 IL-6 in blood are likely to reduce the risk of developing severe COVID-19. Further studies are warranted to support the feasibility of using this finding in prognosis or preventive strategies and to pinpoint the exact protective mechanisms. Further studies will need to clarify whether the protective role against a severe disease course of higher baseline pre-infection levels of IL-6 extends to other infectious diseases.

## Data Availability

The COVID-19 GWAS data analyzed here can be accessed through HGI consortium website (https://www.covid19hg.org/) as well as GenOMICC data portal (https://genomicc.org/data/). All SNP-exposure associations, their source GWAS studies, and the results of all MR and sensitivity analysis are provided in Supplementary Tables.

## Supplementary Materials

The following supporting information can be downloaded at: https://www.mdpi.com/article/10.3390/genes15070889/s1, Figure S1: leave-one-out MR analysis of IL-6 and sIL6ST on severe COVID-19; Table S1: Genome-wide association studies (GWAS) of which instruments were extracted to be used in MR analyses; Table S2: SNPs associated with IL-6 pathway components that were used as Instrumental variables in MR analyses; Table S3: MR results of the main and sensitivity analyses for IL-6 sigaling components against COVID-19; Table S4: Results of heterogeneity and pleiotropy tests in the effects of IL-6 pathway components on severe COVID-19; Table S5: Look up of SNP associations with exposures in independent GWAS datasets; Table S6: Results of multivariable MR, including the effects of IL6, sIL6R, and CRP on severe COVID-19; Table S7: STROBE-MR Checklist of Recommended Items to Address in Reports of Mendelian Randomization Studies.
