# Supplementary figures and images for: Pathway-Based Mendelian Randomization for Pre-Infection IL-6 Levels Highlights Its Role in Coronavirus Disease
Source: Genes (Basel). 2024 Jul 6;15(7):889. doi: 10.3390/genes15070889 (PMC11275426; doi:10.3390/genes15070889)

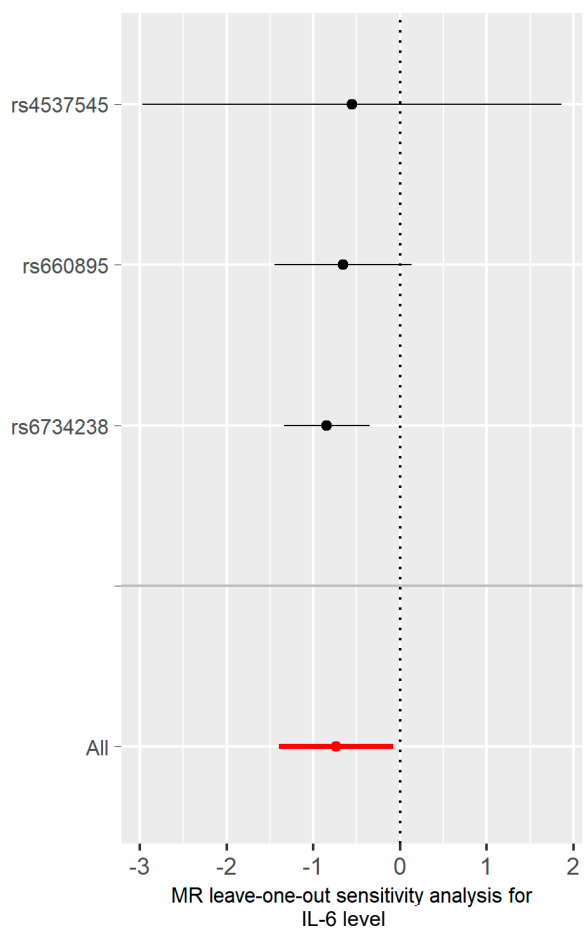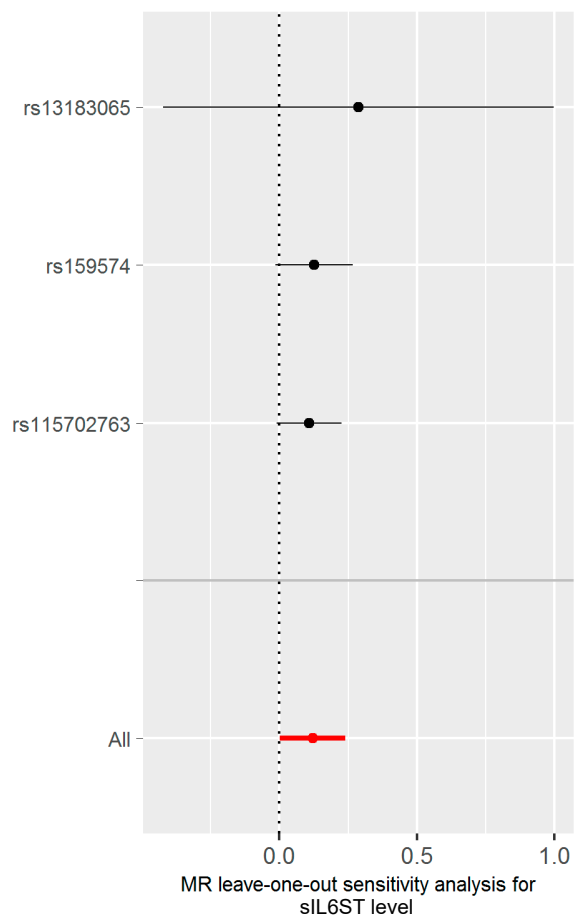

**Figure S1.** leave-one-out MR analysis of IL-6 and sIL6ST on severe COVID-19.

Supplement: Supplementary file 1 [file genes-15-00889-s001.zip › Figure S1.pdf]
